# A HTML5 open source tool to conduct studies based on Libet’s clock paradigm

**DOI:** 10.1038/srep32689

**Published:** 2016-09-13

**Authors:** Pablo Garaizar, Carmelo P. Cubillas, Helena Matute

**Affiliations:** 1 Facultad de Ingeniería, Universidad de Deusto, Bilbao, Spain; 2 Facultad de Ciencias de la Salud y de la Educación, Universidad a Distancia de Madrid, Madrid, Spain; 3 Facultad de Psicologia y Educación, Universidad de Deusto, Bilbao, Spain

## Abstract

Libet’s clock is a well-known procedure in experiments in psychology and neuroscience. Examples of its use include experiments exploring the subjective sense of agency, action-effect binding, and subjective timing of conscious decisions and perceptions. However, the technical details of the apparatus used to conduct these types of experiments are complex, and are rarely explained in sufficient detail as to guarantee an exact replication of the procedure. With this in mind, we developed Labclock Web, a web tool designed to conduct online and offline experiments using Libet’s clock. After describing its technical features, we explain how to configure specific experiments using this tool. Its degree of accuracy and precision in the presentation of stimuli has been technically validated, including the use of two cognitive experiments conducted with voluntary participants who performed the experiment both in our laboratory and via the Internet. Labclock Web is distributed without charge under a free software license (GPLv3) since one of our main objectives is to facilitate the replication of experiments and hence the advancement of knowledge in this area.

Humans constantly interact with their environment. Picking up the phone and pressing a computer key are actions that people perform almost unconsciously in their daily lives. These actions have an observable effect; the phone stops ringing or a character appears on the screen. Thus, people are active agents in the control of events in their environments.

The study of how people detect that they are agents of certain effects, and of how they perceive the relationship between their actions and their potential effects has been an interesting and productive research topic since the early times of experimental psychology. Among the questions that have been explored are the perception of the causal relationship between an action and its effect, consciousness and willingness to initiate voluntary acts, as well as the perceived temporal distance between actions and their consequences.

Wilhelm Wundt had already reported the seminal experimental work in this area as early as 1887 (see^1^). He designed a complex apparatus in which a clock’s hand rotated through a sphere, with the occasional presentation of an auditory stimulus. Wundt asked his experimental participants to indicate the position of the clock hand when the tone was presented. In this way he could investigate whether the participant’s subjective perception of the tone coincided with its objective timing.

This methodology was then adapted in the twentieth century by Libet and his colleagues, who used an oscilloscope with a dot rotating around a sphere^2^. We will refer to this method as Libet’s clock. Variations of this procedure (including current computerized versions), have been used to investigate a number of interesting research phenomena in psychology and neuroscience^3^,^4^,^5^,^6^,^7^,^8^. For instance, using Libet’s clock, Haggard and his colleagues asked their experimental participants to press a key in a computer keyboard anytime they wished while a dot was rotating around a sphere in the computer screen^3^. Upon pressing the key, a 250-millisecond delay was followed by the presentation of a tone. Participants were asked to estimate the position of the dot at two different moments - either when the tone sounded or when they pressed the key. The results showed that when participants were asked to estimate the dot’s position at the time of the key press, there was a forward shift in this estimation (that is, they judged the key press had occurred later in time). However, when participants were asked to estimate the position of the dot when the tone sounded, participants judged that it occurred earlier than the time of its actual onset. The misjudgment of the dot’s position in both situations is known as *temporal binding*, a subjective reduction of the temporal delay between the two events. In other words, the action and its consequence are perceived as being closer to each other than they actually are. This effect has also been called *intentional binding*^3^ and it is the subject of intense theoretical debate^9^,^10^: Some researchers have argued that it is causation, rather than intentionality, that is critical in producing this phenomenon^11^, while others contend that both intentionality and causality are critical^12^. Interestingly, it has also been shown that when people get used to a certain delay between their actions and their effects, presenting the effects with a shorter delay may even reverse the perception of the events, so that the tone might be perceived as occurring before the action^13^. Other “time reversal paradoxes” have been described in the literature, including Warren’s phonemic restoration effect^14^, which also works “backwards” in time by restoring earlier missing information in a heard sentence based on later-arriving information. There is a large class of perceptual restoration effects, including the famous blind spot phenomena where we routinely fill in visual field percepts in the scene, when that patch of input is actually a scotoma induced by the missing retinal receptors where we might expect receptors to exist^15^. These are not so much “illusions” as “restorations” of the expected perceptual content, in spite of missing input information

Using Libet’s clock together with electroencephalographic (EEG) recording, a delay in the conscious experience of the perception of a stimulus has also been reported^16^, along with the timing of conscious decisions^2^. In those experiments, the participants’ brain activity was monitored while they were asked to estimate the position of the dot in a Libet’s clock when they “felt the will” to press a key, when they pressed it, or when a tone sounded after their key-press. The results showed that their brain activity was first in the temporal chain, with their “feeling the will” to act occurring after their brain activity had already started, and their perception of the action following their “feeling the will”. Their conscious perception of the sound closed the chain^2^,^16^. These experiments have fueled a very interesting debate on the notions of conscious will and sense of agency, suggesting that conscious will might be a by-product of brain activity.

Regardless of the merits of the various theoretical interpretations and debates that have flourished around these experiments using Libet’s clock, and even though some alternative procedures have also been developed (for alternative proposals see^1^,^17^,^18^), it is clear that Libet’s clock is still a popular experimental paradigm that is used to study a number of interesting questions in psychology and neuroscience, such as the perception of external stimuli like tones, and internal cues such as the sense of agency and the timing of conscious decisions.

To the best of our knowledge, however, there is not a standardized version of Libet’s procedure that could be simply programed to fit one’s experiment. Such version would greatly facilitate replicability and homogeneity and should probably not be expensive to use in today’s computerized laboratories. By contrast, as we have been able to learn, each research group seems to have developed their own software over the years, which makes this procedure difficult to share among researchers (we were not able to obtain a copy), due to the “home-made”, personal, features of most current versions. In addition, little technical details are typically provided with manuscripts, which, given the extreme precision and accuracy that are required in timing studies, makes replicability difficult and opens the doors to potential software and timing errors (both at the operating system level, which may be open to timing issues, and at the level of the programming language used to develop the experimental paradigm). Even though it is possible that those potential errors are absent in most previous research in the literature, one cannot discard their existence when the articles do not provide the necessary details for code inspection and accurate reproducibility of the experiments.

Thus, having a public and standardized version of Libet’s clock which could be used freely to conduct experiments on sense of agency, intentional binding, and related phenomena should facilitate enormously the advancement of knowledge in these areas, the development of new experiments and the replicability of findings. With this in mind, we developed Labclock Web, a tool that researchers could use freely to conduct experiments. Labclock Web offers three main improvements when compared with current tools used to conduct experiments with Libet’s procedure. First, Labclock Web is open source software. It is free and is highly flexible, as its code is public and can be adapted according to the aims of each experiment (e.g., it might be connected to additional apparatus). If errors are present, anyone could detect them, report them, and solve them. Second, using external configuration files, experimental tasks are easily programmed by non-expert programmers. Although some basic computing skills are required, tasks can be adapted to each experimental situation without extensive knowledge of programming. Finally, Labclock Web operates in web browsers, a feature which allows for experiments to be conducted online.

Labclock Web takes advantage of the latest web standards to provide a multi-platform application for conducting online experiments. Below we describe the technical features of this tool and the tests that we have conducted to guarantee the accuracy of stimulus presentation–an issue of critical importance in experiments of this sort. In addition, and because the potential problems in running these experiments arise not only from technical aspects, but also from the behavioral ones (particularly in those experiments conducted through the Internet), we also conducted two experiments on intentional binding with human participants. The first one was conducted in the laboratory, whilst the second was carried out online. The results confirmed that Labclock Web is a reliable tool for conducting experiments in this area. They also show that reliable experiments can be carried out online but that some additional cautionary measures need to be taken into account in those cases.

## Libet’s Clock Basic Procedure

**Figure 1:**
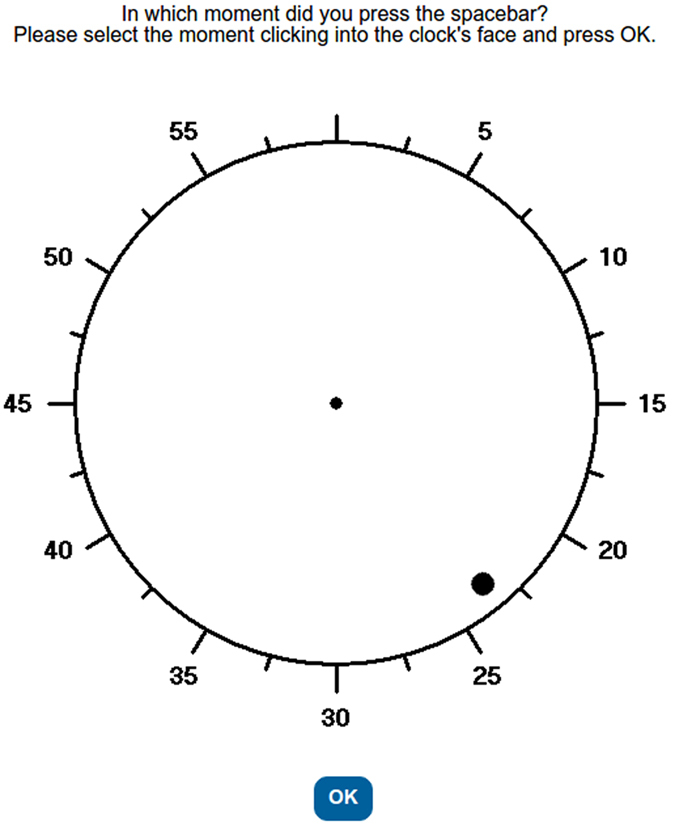
Screen capture showing the requirement for emitting an action judgment at the end of each trial in Labclock Web.

As could be imagined, there are important differences between the procedure developed by Wundt in the 1880s, the one developed by Libet and his colleagues in the 1980s, and the many computerized versions developed in the 2000s (e.g.^3^,^4^). But despite those many variations, the general, basic procedure used today, can be summarized as follows: (1) at the beginning of each trial, a warning message tells participants to be prepared; (2) after a delay of variable duration (typically between 1000 and 3000 ms), a clock face is shown; (3) instead of clock hands, a dot starts to spin around the center of the clock face at a constant speed (typically 2560 ms per cycle); (4) during the first round of the dot’s rotation, participants must not take any action (the purpose of the first round is that they become used to the speed of the dot); (5) during the second round of rotation, participants are free to press a key whenever they wish (t = T_key_); (6) once the second round of the rotating dot has finished, an empty clock face is shown to participants and they are asked to indicate the moment they decided to press the key (t = T_will_). A further dependent variable that is often reported is the moment at which participants believe that they pressed the key (t = T_action_)^2^,^16^. We will call these judgments *action judgments*. Figure 1 shows how they were collected in our experiments. Furthermore, in some studies researchers provide immediate vs. delayed auditory feedback after the key press to study its influence on the participant’s timing perception of the action^3^ or the decision to act^4^. In our experiments we will also use of this strategy.

## Underlying Technologies of Labclock Web

Considering the strict timing requirements of Libet’s paradigm, we have developed Labclock Web using new HTML5 Application Programming Interfaces (APIs) that maximize accuracy and timing precision. These include the following features: 1) CSS Animations for presenting visual stimuli, 2) Web Audio API for presenting auditory stimuli, and 3) DOM event timestamps for logging user interaction. Labclock Web performs several sanity checks through Modernizr library to prevent experiments from being conducted on platforms not compliant with these HTML5 APIs. Similarly, Labclock Web checks whether the display resolution is suitable for adequate presentation of the stimuli. If any of these checks fail, error messages are shown.

In Labclock Web, the rotating dot animation of Libet’s clock operates by means of a CSS animation. As shown in Listing 1, the spin animation has two key frames associated with rotation transformations. Thus, web browsers will rotate the element with id = dot from 0 to 360 degrees (i.e., a complete cycle). The initial state of the animation (animation-play-state) is paused since it is initiated by Labclock Web at the beginning of each trial. Since two complete turns are required in each trial, the number of iterations in the animation is 2 (animation-iteration-count). The rotating dot will have a constant angular velocity due to the fact that the selected timing function (animation-timing-function) is linear. The dot’s animation duration is typically set to 2560 ms and the initial delay (animation-delay) can be modified depending on the configuration of each trial. The definition of the rotating dot animation shown in Listing 1 does not take into account all vendor prefixes needed to run the animation in older versions of web browsers (i.e., webkit for Google Chrome and Apple Safari, moz for Mozilla Firefox, ms for Internet Explorer, o for Opera, etc.), but Labclock Web includes them for compatibility reasons.

With respect to auditory stimuli, Labclock Web relies on the start function of Web Audio API, which takes one argument: the number of milliseconds the audio play will be delayed. This value is defined in the experiment’s configuration file. In order to avoid unexpected delays, Labclock stores all auditory stimuli in buffers at the beginning of the experiment.

The timestamps of participant key-presses are recorded using DOM Events, which provide, in milliseconds, a timestamp of the creation of the event (i.e., animationStart, keypress). Using High Resolution Timer API (i.e., window.performance.now function) at the beginning of event handler functions would improve the resolution of the measurement (this API provides microsecond resolution), whilst accuracy and precision are lower because these timestamps would be collected not at the moment the events were created, but when their event handlers were executed.

Once the experiment has been completed, Labclock Web sends the results through a HTTP Post connection via AJAX. Additionally, a local backup will be stored in the user’s browser using the Local Storage API (useful for experiments conducted in a laboratory with no Internet connection or for recovering participant’s data when Internet connection-related problems occur). The format of the results stored by Labclock Web is detailed in Table 1 (see details in the “Configurable parameters” section). Having completed the development of Labclock Web, we considered it necessary to assess the degree of compliance with its strict time requirements. Given the nature of the rotating animation needed in Libet’s paradigm, it has not been possible to use standard procedures for analyzing the accuracy and precision of the presentation of visual stimuli (e.g., Black Box Toolkit; see^13^). These procedures are based on the detection of the onset time by photosensors^13^. However, the circular path followed by the rotating point of Libet’s clock produces difficult–to–interpret patterns in photosensors, with rapid fluctuations from white to black when the rotating dot approaches the photosensor. For this reason, we used a high-speed camera (CASIO ZR-100) capable of recording video clips at 1000 FPS (frames-per-second).

**Figure 2:**
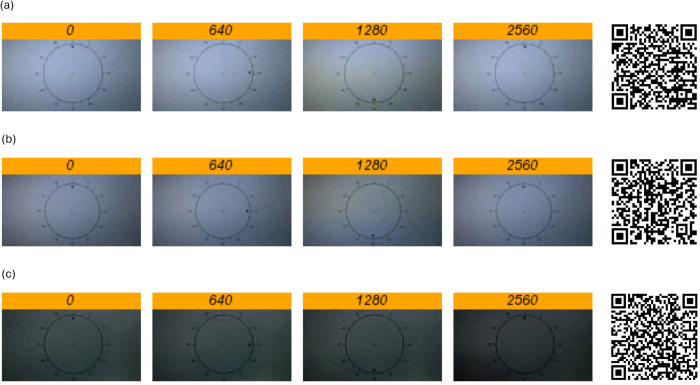
Key frames of Labclock Web animations captured through a high-speed camera: (**a**) Labclock Web on Google Chrome under Windows, (**b**) Labclock Web on Google Chrome under GNU/Linux, (**c**) Labclock Web on Google Chrome under Mac OS X.

Labclock Web has been tested with Google Chrome for Windows, GNU/Linux and MacOS X. We chose this browser due to its high market share and its proper implementation of the underlying technologies (particularly with regard to the Web Audio API, designed by Google). For each of the Operating Systems mentioned, a high-speed video was recorded. Given that the videos were recorded at 1000 FPS, each frame represents a millisecond. Therefore, we extracted each frame from these videos and labeled them with a number. We then created a GIF89a animation with all labeled frames to recreate the movement of the rotating dot in Labclock Web for each case. We decided to use GIF89a since, unlike some video codecs, this format does not perform optimizations between similar frames, which prevents occasional frame loss or overlap. Figure 2 shows the key frames of the Labclock Web tests in Google Chrome on Windows, GNU/Linux and MacOS X. QR codes linking to these GIF89a animations are also provided. As can be seen, the period of 2560 ms configured in Labclock for each Operating System is properly fulfilled in all cases.

As mentioned previously, Labclock Web relies on the Web Audio API to present auditory stimuli accurately. To prevent unwanted delays due to the preparation of the auditory stimulus, Labclock Web generates the tone procedurally and stores it in memory using a BufferSourceNode. The accuracy and precision of this technology was measured by an auxiliary computer connected using an audio cable to the computer that runs the experiment. This auxiliary computer was provided with a GNU/Linux distribution, which specializes in low-latency audio recording (AVLinux with a real-time Linux kernel with IRQ Threading support). This setup is able to record audio with a latency of 1.68 ms, a value comparable to professional digital audio consoles. Once we had prepared the testing setup, we used the Web Audio API to generate a sequence of 1 kHz tone for 200 ms followed by 800 ms of silence. For each Operating System (Microsoft Windows, GNU/Linux, MacOS X) we measured 5 independent sequences and took the first 100 samples from each sequence. Thus, 500 measurements for each Operating System were analyzed. Table 2 clearly shows that this way of generating auditory stimuli on the Web is accurate and precise in all Operating Systems analyzed, with sub-millisecond means and SDs. In order to contextualize these results, the same procedure was carried out using the DMDX experimental software^19^. As expected, DMDX was also able to accurately present auditory stimuli, with an average delay of −1.445 ms (i.e., on average, an auditory stimulus was presented 1.445 ms before expected) and an SD of 3.214 ms. Considering the expected audio recording latency (1.68 ms), both Web Audio API and DMDX provided solid results.

### Setting up experiments with Labclock Web

One of the main goals of Labclock Web is to facilitate its use by researchers who have no programming skills. Therefore, setting up an experiment for Labclock Web can be accomplished by editing configuration files using a text editor. This section explains how to create or modify these configuration files to get the most out of Labclock Web.

Labclock Web’s experiment execution flow starts by presenting a set of initial screens (these are typically used for welcome, informed consent, and instructional information). Once participants have read all initial screens, they are asked to enter a passcode. This is useful to prevent unwanted participants from taking part in the experiment. Further, in the case of an offline experiment, it allows researchers to ensure that all participants understand the instructions and all of them begin at the same time. Labclock then conducts all the experimental phases defined in the configuration file. Each experimental phase is defined by a set of trials and a final information screen. At the beginning of each trial, Labclock Web plays a tone to warn participants. The rotating dot of Libet’s clock then begins to spin and participants’ responses are recorded. Once all the trials of an experimental phase are shown, its final screen is displayed (some researchers use this screen to instruct participants about details of the following phase). When there are no more experimental phases to be conducted, Labclock Web presents a set of final screens (these are typically used to debrief the participants concerning the purpose of the study). Finally, the data is locally stored and sent via a HTTP Post connection.

### Configurable parameters

Experiment configuration files in Labclock Web are defined in JSON (JavaScript Object Notation). A sample configuration file is shown in Listing 2 (the ellipses indicate omissions made, to facilitate understanding of the format). To define different groups or experimental conditions, different properties of the experiment object should be defined. In this case, property A represents Group A in the experiment. Each of these experimental conditions allows for the possibility of having different configurations within the same experiment and we can define as many as are necessary. Participants can be assigned to each condition manually or pseudo-randomly (using selectExperiment with false or true arguments, respectively).

For each group or experimental condition there are some general properties that need to be be defined: 1) code, to indicate the name or code of the experiment; 2) password, to set the passcode to protect the beginning of the experiment; 3) randomDelayMin and randomDelayMax, to define the minimum and maximum values (in ms) of the random delay that precedes each trial (use the same value in both for a constant delay); 4) postResultsURL, to indicate the URL where the results will be sent at the end of the experiment; 5) responseKey, to indicate which key is considered the answer key; 6) sounds, to define the sounds used in the experiment (the getReady tone is defined by a path to an audio file, and the feedback tone is defined by a frequency and duration, since this tone is procedurally generated by Web Audio API); 7) messages, to define messages during trials and to localize / translate the experiment; 8) preScreens, a set of screens (title and content for each one) to be presented at the beginning of the experiment; 9) passwordScreen, to define the screen where the passcode is requested; 10) phases, an array of objects representing each phase (explained later); and 11) postScreens, a set of screens to be presented at the end of the experiment.

Each of the objects in the phase array defines an experimental phase. These are the configurable parameters in each phase: 1) description, a brief text to describe the phase (not shown to participants, only for internal use); 2) progress, a boolean value to enable or disable a progress bar during trials; 3) scramble, a boolean to scramble all trials of this phase using the Fisher-Yates shuffle; 4) trials, a set of trials defined by their cycle (period of the rotating dot in ms, typically set to 2560) and tone (delay in ms for the feedback tone; if this property is not defined, no feedback is provided on that trial) properties; and 5) screen, a final screen to provide information about this phase or the next phase. For every trial, Labclock Web stores all variables described in Table 1.

### Overview of the experiments

In addition to testing the technical accuracy of Labclock web, and because the potential problems in running these experiments involve not only technical aspects, but also behavioral ones (particularly if the experiments are run through the Internet), we also conducted two additional tests with human participants. Experiment 1 was conducted in the laboratory, Experiment 2 was conducted online.

One area where we thought that would be highly interesting to test the accuracy of Labclock Web both offline and online was intentional binding^3^. In principle, if we provided auditory feedback following participants’ actions, then participants’ subjective perception of their action should be misplaced towards the time when the auditory feedback was presented. Thus, by using in Experiment 1 an immediate versus a delayed auditory feedback in randomly selected trials we could test whether the perception of the action was misplaced. Moreover, it could be particularly interesting to test this effect in an online setting in Experiment 2. It has been shown that previous biases and expectations have an important role in the development of these effects^10^, and when interacting with web applications people often have the expectancy (which is often correct) that things will work slower^20^,^21^. Thus, they should expect a delay between their actions and their effects. Moreover, it has been shown that when people expect the effect to occur at a given time and it occurs at a different time, then the sense of agency is reduced^9^ and the perception of the events can even be reversed^13^. Thus, it could be that even though we solved the technical aspects of Labclock Web as described above and ensured that the stimuli occurred through the Web at the precise time when they were supposed to occur, if people had these expectations of everything being slower through the Web, their sense of agency might be reduced and their judgments influenced by these expectations.

In order to control for the potential effect of people’s expectations of the action-effect delay on the Internet, we also assessed their baseline timing judgments for their action when no external effects were presented. Baseline judgments are often^3^,^7^ (though not always) used in this type of experiment; we believe that their use is essential in cases like the present experiment in which biases are highly likely to occur.

Thus, in both experiments we used two different action-effect intervals (1 vs. 500 ms) which were presented randomly through the training sessions, and we then added a baseline phase at the end of each experiment. In this baseline phase we did not present any auditory feedback. Its purpose was to record a baseline of the subjective judgments that the participants had for the timing of their actions (in the absence of any external feedback) both offline and online. Therefore the dependent variable of interest was computed as the difference between the mean action judgments that participants provided for the timing of their actions in each condition (i.e., immediate vs. delayed feedback) and the mean baseline judgments.

### Ethics statement

The computer program informed participants that their participation was voluntary and anonymous. We did not ask participants for any data that could compromise their privacy, nor did we use cookies or software in order to obtain such data. The stimuli and materials were harmless and emotionally neutral, the goal of the study was transparent, and the task involved no deception. According to the U.S. Department of Health and Human Services (2009; Section 46.101b)^22^, as well as the American Psychological Association (2002; Section 8.05)^23^, no written informed consent is required under these circumstances. Therefore we did not collect them so that volunteers did not need to identify themselves. The ethical review board of the University of Deusto examined and approved the procedure used in this research. The two experiments were conducted in accordance with the approved guidelines.

### Experiment 1

#### Participants

This experiment was conducted in the Experimental Psychology laboratory at the University of Deusto, with the voluntary participation of students that were rewarded economically. A total of 64 participants took part in the study. Data from 7 of them were discarded due to not answering more than 25% of trials of either one of the two conditions.

#### Apparatus and materials

Booths in the Experimental Psychology laboratory of the University of Deusto, equipped with Personal Computers, were used to conduct the experiment. These computers ran Labclock Web on a Google Chrome browser in Microsoft Windows 7. Auditory stimuli were presented to participants via headphones connected through the audio output of the PCs.

#### Procedure

The design of this experiment was a within-subject design with two types of trials: Those in which the tone that follows the action of the subject sounds immediately (1 ms) and those where the tone was delayed (500 ms). Each participant was presented with 40 trials for each condition, intermixed using a predefined pseudorandom sequence. To prevent a possible effect of the particular sequence, two different pseudorandom sequences were used and participants were randomly assigned to one of these. Immediately after each one of the 80 trials, participants were asked to indicate the time at which they believed they had pressed the key (T_action_) on that trial. Finally, after all 80 trials (40 with immediate feedback, 40 with delayed feedback) had finished, participants were presented with 20 additional trials with no feedback in order to establish the baseline for their action judgments when no feedback was given in this experiment. Taking into account the results of previous studies and the design described herein, our hypothesis was that the T_action_ for immediate feedback trials would be significantly lower than for delayed feedback trials, particularly after we had adjusted for the baseline judgments of each participant.

#### Results and discussion

**Figure 3:**
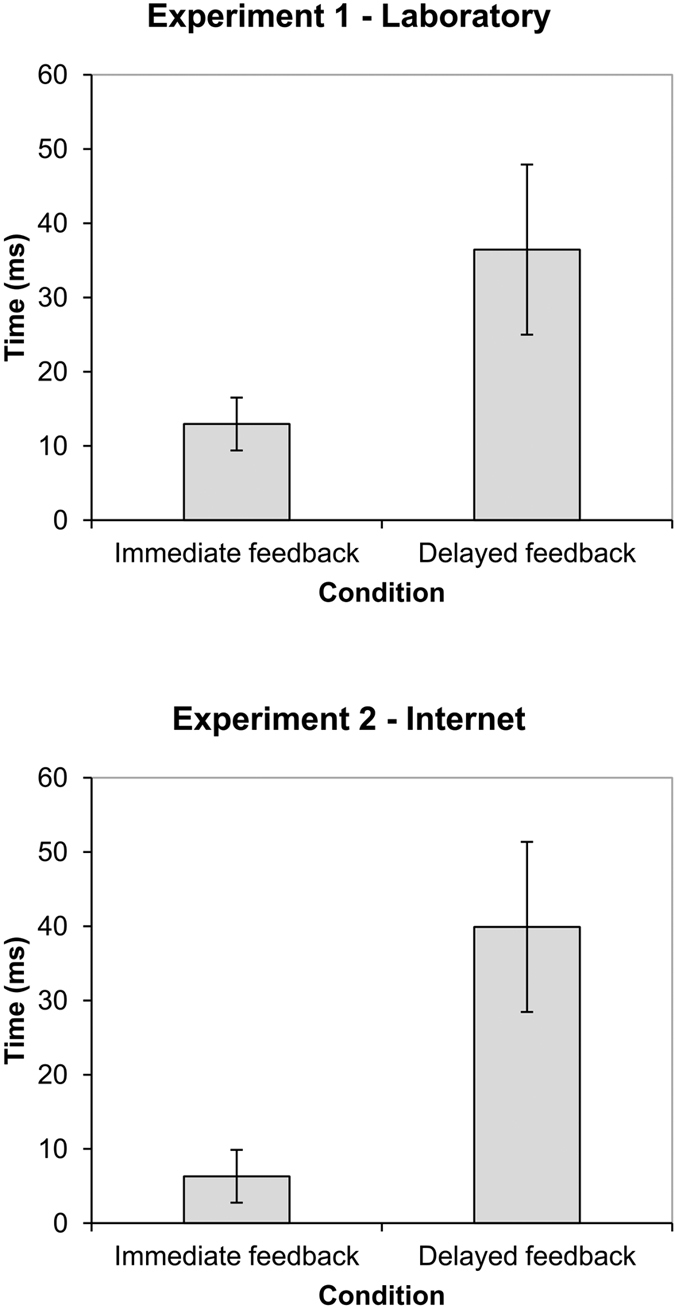
Mean action judgments in immediate (1 ms) and delayed feedback (500 ms) using Labclock Web. Judgments are shown in ms (after subtracting baseline judgments), with 0 ms being the time at which the action actually occurred. The top panel shows the results of Experiment 1, conducted in the Laboratory. The bottom panel shows the results of Experiment 2, conducted via the Internet. Error bars represent the standard error of the mean.

For each participant we computed the difference between T_action_ and mean baseline judgment in each of the feedback conditions (immediate vs. delayed). The top panel of Fig. 3 shows the means and SEM for the immediate (1 ms) feedback and delayed (500 ms) feedback conditions in this experiment. The results are given in ms from the actual time of responding (i.e., 0). As can be seen in the figure, the results were consistent with our hypothesis. That is, action judgments were larger in the delayed feedback condition than in the immediate feedback condition. These two conditions were compared using a related samples t-test which revealed a significant difference between them, *t*(56) = 2.459, *p* = 0.017, *d* = 0.46.

In addition, and in order to make sure that these findings did not depend on the use of the baseline judgments, we also analyzed the raw judgments (i.e., without baseline subtraction). Although the actual numbers are slightly different, the conclusions do not vary: Judgments in delayed feedback condition (M = 41.152, SEM = 12.936) are significantly larger than judgments in the immediate condition (M = 3.806, SEM = 6.136), *t*(56) = 3.513, *p *< 0.001, *d *= 0.66.

The results are in accord with our hypothesis. That is, when the consequence of participants’ response (i.e., feedback) was delayed, they estimated the timing of their key press to be later, compared with the case in which there was no delay.

### Experiment 2

#### Participants

Experiment 2 was identical to Experiment 1, with the exception that the 55 participants were recruited online. Data from 3 participants were eliminated using the same criteria as in Experiment 1 (i.e., not answering more than 25% of trials in one of the two conditions).

#### Apparatus and materials

Participants ran Labclock Web on their own personal computers through the Internet using Google Chrome browser (since this browser was the only one which implemented the Web Audio API when the experiment was conducted) on all major Operating Systems (Microsoft Windows, GNU / Linux and Apple MacOS X).

#### Procedure

The procedure of this experiment was the same as that described for Experiment 1 except that it was conducted through the Internet.

#### Results and Discussion

The results were analyzed as in the previous experiment. That is, for each participant we computed the difference between T_action_ and mean baseline judgment in each condition. The bottom panel of Fig. 3 shows the means and SEM for the immediate (1 ms) feedback and delayed (500 ms) feedback conditions in this experiment. The results are shown in ms from the actual time of responding (i.e., 0). As can be seen in this figure, the mean action judgments were larger in the delayed feedback condition than in the immediate feedback condition. These two conditions were compared using a related samples t-test which revealed a significant difference between them, *t*(51) = 2.372, *p *= 0.021, *d *= 0.47. Thus, the results are consistent with our hypothesis that the T_action_ for delayed feedback (500 ms) trials should be significantly larger than that for immediate feedback (1 ms) trials.

In addition, and in order to make sure that these findings did not depend on the use of the baseline judgments, we also analyzed the raw judgments (i.e., without baseline subtraction). The actual numbers are different, but the main findings are similar: Judgments in the delayed feedback condition (M = 0.041, SEM = 15.868) are significantly larger than judgments in the immediate condition (M = −32.967, SEM = 7.786), *t*(51) = 2.647, *p *= 0.01, *d *= 0.52. Interestingly, these raw data from the Internet experiment suggest that, as we expected, this experiment was sensitive to the participants’ expectation that things work slowly online. Thus, in the immediate feedback condition, in which the tone occurred immediately (1 ms) after the action, participants inferred that their action must had occurred earlier than it actually did. This finding is consistent with the existence of other time reversal findings in the literature^13^,^14^ and with the critical role of previous biases in the development of these effects^10^. This finding also supports our suggestion that it is important to compensate for these biases using the baseline judgments in each experiment, especially when the experiment is conducted through the Internet and therefore strong biases and expectations are present.

## General Discussion

As part of the technological revolution, the last decade has witnessed notable developments in the World Wide Web. Many of the activities that were typically performed by offline desktop applications have been replaced by web applications running in the browser (e.g., email, office, and even multimedia edition). The enormous benefits of running on a connected execution environment in which technology requirements can be met on demand (in Cloud Computing all is offered “as a Service”) outweigh the disadvantages associated with the loss of performance of web applications against native applications. However, the emergence of increasingly sophisticated and optimized web APIs is reducing these differences in performance at an astounding rate, and the large number of mobile applications that have been created using development platform frameworks (e.g., Cordova) serves only to confirm this trend.

This paradigm shift in which web applications based on open standards such as HTML5 replace offline desktop applications is also happening in the field of Psychology and Neuroscience research software^24^,^25^,^26^. This is due not only to technical reasons (i.e., running experiments with no installation costs, multi-platform experiments, and so on), but also to methodological issues, since the same application can be used for experimentation both in the laboratory and online. Most importantly, it has the potential to serve as a standardized methodology that can facilitate the reproducibility of research results.

In this article we presented Labclock Web, a web tool to perform online experiments using Libet’s clock. We conducted both technical tests of the accuracy of this tool and behavioral tests, in order to make sure that it was suitable for use in the laboratory (Experiment 1) and on the Internet (Experiment 2). The results are consistent with our hypothesis that the T_action_ for delayed feedback trials should be significantly larger than that for immediate feedback trials. This was shown in both the online and the offline. This effect replicates and extends findings of many previous experiments in this area. For instance, Banks and Isham^4^ observed very similar results using a different question and intervals that ranged from 5 to 60 ms, and Haggard and his colleagues^3^,^7^ observed similar results using an action-tone interval of 250 ms. In our experiments, we observed the same effect with longer-than usual intervals (i.e., 500 ms), which should be easier to implement, and the effect size is within normal values for this type of experiments^27^. Therefore, the accuracy and precision of Labclock Web is supported not only through the technical tests that we described in previous sections but also through these two behavioral experiments. In addition, this innovation now allows for research using Libet’s clock to be conducted online, which represents an unprecedented achievement

In addition to our own research, one of our main objectives is to facilitate the replication of experiments and hence the progress of research conducted in this area. Therefore, Labclock Web is distributed without charge under a free software license (GPLv3). This not only allows any researcher to adapt the software to their needs, but also ensures that psychology and neuroscience software developers can share their improvements, corrections of any unnoticed errors that might remain, or derivative versions of Labclock Web with consequent benefit to the whole scientific community. Some 30 years later, Libet’s clock is now available on the Web. Our greatest wish is that researchers worldwide will take the opportunity to exploit this tool to their advantage, and that improving the methods for studying the human sense of agency and consciousness will help us to move forward in our understanding of these topics.

### Data availability

The code of Labclock Web can be downloaded from its public repository: https://github.com/txipi/Labclock-Web.

## Additional Information

**How to cite this article**: Garaizar, P. *et al*. A HTML5 open source tool to conduct studies based on Libet's clock paradigm. *Sci. Rep.* **6**, 32689; doi: 10.1038/srep32689 (2016).

## Supplementary Material

Supplementary Information

## Figures and Tables

**Table 1:** Recorded variables for each trial in Labclock Web.

Name	Description
InitialRandomTime	Initial delay time (in ms) before the clock starts spinning (we can set it using randomDelayMin and randomDelayMax properties in the setup file).
Cycle	Configured duration (in ms) of a spinning dot cycle for this trial.
CycleTime	Measured duration (in ms) of the two spinning dot cycles for this trial.
Tone	Configured delay (in ms) of the auditory stimulus for this trial.
ToneTime	Measured delay (in ms) of the auditory stimulus for this trial.
KeyPressTrialTimes	Comma-separated list of timestamps (in ms) of all space bar key presses during the whole trial.
StartTrialTime	Timestamp (in ms) of the beginning of the trial.
EndTrialTime	Timestamp (in ms) of the end of the trial (EndTrialTime –StartTrialTime = duration of the trial).
StartTrialAudioTime	Timestamp (in s) of the beginning of the trial using the currentTime property from Web Audio API.

**Table 2:** Mean and SD of auditory stimuli presentation timing errors using web Audio API on Google Chrome (in ms).

OS	Mean	SD
Windows	−0,44526	0,542153
GNU/Linux	−0,41315	0,726393
Mac OS X	−0,44522	0,204533
