# Supplementary material for: A HTML5 open source tool to conduct studies based on Libet’s clock paradigm
Source: Sci Rep. 2016 Sep 13;6:32689. doi: 10.1038/srep32689 (PMC5020737; doi:10.1038/srep32689)
Supplement: Supplementary Information [file srep32689-s1.pdf]

A HTML5 open source tool to conduct studies based on Libet's clock paradigm

Pablo Garaizar*[1], Carmelo P. Cubillas[2], & Helena Matute[3]

[1]*Facultad de Ingeniería, Universidad de Deusto, Bilbao, Spain*

[2]*Facultad de Ciencias de la Salud y de la Educación, Universidad a Distancia de Madrid, Madrid, Spain*

[3]*Departamento de Fundamentos y Métodos de la Psicología, Universidad de Deusto, Bilbao, Spain*

Mailing address:

Pablo Garaizar

Facultad de Ingeniería

Universidad de Deusto

Avda. Universidades 24, 48007, Bilbao, Spain.

Tel: +34 944 139 312

e-mail: garaizar@deusto.es

Listing 1

Rotating dot CSS Animation in Labclock Web.

```
#marks #dot {
  animation-play-state: paused;
  animation-name: spin;
  animation-iteration-count: 2;
  animation-timing-function: linear;
  animation-duration: 2.560s;
  animation-delay: 0.5s;
}

@keyframes spin {
  from { transform: rotate(0deg); }
  to { transform: rotate(360deg); }
}
```

Listing 2

Labclock Web configuration file example (summarized).

```
experiment.A = {
  code: 'Binding A',
  password: '99',
  randomDelayMin: 1000,
  randomDelayMax: 3000,
  postResultsURL: 'datasent.asp',
  responseKey: ' ',
  sounds: {
    getReady: { file: 'media/250-440Hz_44100Hz_16bit_1000ms.wav' },
    feedback: { duration: 200, pitch: 1000, }
  },
  messages: {
    commandOK: 'OK',
    ...
  },
  preScreens: [
    { title: '...', content: '...' },
    ...
  ],
  passwordScreen: { title: '...', content: '...' },
  phases: [
    {
      description: 'Binding',
      progress: true,
      scramble: true,
      trials: [
        { cycle: 2560, tone: 1 },
        ...
        { cycle: 2560, tone: 500 },
        ...
      ],
      screen: { title: '...', content: '...' }
    },
    {
      description: 'Post base rate',
      progress: true,
      scramble: false,
      trials: [
        { cycle: 2560 },
        ...
      ],
      screen: { title: '...', content: '...' }
  ],
  postScreens: [
      screen: { title: '...', content: '...' }
  ]
};
```
